# Spontaneous conversations as data in young-onset dementia research: a qualitative study

**DOI:** 10.3389/frdem.2026.1805579

**Published:** 2026-04-29

**Authors:** Martina Quartarone, Ludovica Forte, Lara Calabrese, Marco Brigiano, Alice Annini, Sara Trolese, Giorgia Vella, Francesca Vaienti, Manuela Boschetti, Rabih Chattat

**Affiliations:** 1 Department of Psychology, University of Bologna, Bologna, Italy; 2 ASP Valloni Marecchia, Rimini, Italy

**Keywords:** co-production, living experience, participatory research, spontaneous conversations, subjective experiences, young-onset dementia (YOD)

## Abstract

**Background:**

Young-onset dementia (YOD), defined by symptom onset before the age of 65, profoundly impacts personal identity, work, and family life. Despite growing interest in the psychosocial dimensions of YOD, the direct voices of people living with the condition remain underrepresented. Existing qualitative research has mainly relied on structured interviews, which can constrain spontaneity and the emergence of authentic meanings. This study adopts an innovative approach by analyzing spontaneous conversations as a naturalistic form of data, enabling a more ecological understanding of living experience.

**Methods:**

An exploratory qualitative study was conducted at an Italian Meeting Center for people with YOD. Eleven participants (aged 55-68) took part in 14 naturally occurring group conversations, recorded between October 2024 and February 2025. Transcripts were analyzed using the *General Inductive Approach*. Themes were co-developed through iterative coding and later validated in a participatory feedback session involving all participants.

**Findings:**

Four overarching themes were identified: (1) *Living with Alzheimer's: between awareness and “broad shoulders”*—redefining identity and meaning in everyday life; (2) *Significant relationships: between closeness, misunderstanding, and new balances*—navigating relational change and finding belonging in peer groups; (3) *The struggle to be believed: between invisible illness and judging gazes*—confronting stigma, disbelief, and social invisibility; (4) *Support that makes a difference: between resources and ideas for living better*—valuing respectful, personalized, and co-designed support. Participants collectively emphasized the need to move from a “for” to a “with” logic in dementia care and research:

“*It is not enough to do something for us. We need to build it together.”*

**Conclusions:**

Spontaneous conversations offer a powerful lens to capture the nuanced, relational, and participatory aspects of living with young-onset dementia. This approach complements traditional qualitative methods by restoring authenticity to the voices of people with dementia, fostering inclusion, and informing the co-construction of more responsive psychosocial and community interventions.

## 1 Introduction

Young-onset dementia (YOD) is defined by the onset of symptoms before the age of 65, a conventional threshold based primarily on sociological criteria rather than on biological or clinical differences compared with late-onset dementia (Rossor et al., 2010). Globally, the age-standardized prevalence of YOD is estimated at 119.0 cases per 100,000 in the 30-64 population, with a progressive increase with advancing age, from 1.1 per 100,000 among those aged 30-34 to 77.4 per 100,000 among those aged 60-64 (Hendriks et al., 2021).

Although the clinical manifestations of dementia may largely overlap regardless of age at onset (Draper and Withall, 2016), YOD often presents with a heterogeneous and non-specific onset, which may include changes in mood and behavior as well as cognitive impairment (Draper and Withall, 2016; Hendriks et al., 2021). This variability contributes to greater complexity in assessment and diagnostic pathways and may delay access to appropriate care and support (Draper and Withall, 2016).

The challenges associated with YOD extend beyond the clinical and diagnostic domain and are particularly significant on the psychosocial level (Arai et al., 2007; Chemali et al., 2012; O'Connor et al., 2007; Sampson et al., 2004). Diagnosis often occurs during a life stage characterized by active employment, family responsibilities, financial stability, and future-oriented plans. This timing may lead to an abrupt disruption of biographical continuity and personal identity (Draper and Withall, 2016; Rabanal et al., 2018; Svanberg et al., 2011). From an occupational and economic perspective, job loss or reduced work participation may undermine financial stability, particularly when the person with YOD was the primary income earner (Evans, 2019; Harris, 2004; Johannessen and Möller, 2013; Kimura et al., 2015; Roach et al., 2016). Consequently, the partner may be compelled to seek additional employment or to reduce working hours in order to meet caregiving demands (Hutchinson et al., 2016; Kinney et al., 2011; Shnall, 2015; Silverstein et al., 2010). At the relational and family level, the impact affects the entire system, particularly the person-partner dyad and, where present, children, requiring a rapid reorganization of roles, functions, and responsibilities, with a potential intensification of emotional and relational burden (Arai et al., 2007; Chirico et al., 2022; Hall and Sikes, 2020). Children may assume additional responsibilities prematurely and experience high levels of emotional stress alongside feelings of loss, anger, frustration, and guilt, with repercussions for physical and psychological wellbeing (Allen et al., 2009; Chirico et al., 2021; Gelman and Rhames, 2018; Hutchinson et al., 2016; Svanberg et al., 2011). Socially, maintaining relationships, community participation, and engagement in meaningful activities is crucial for cognitive and emotional wellbeing (Tonga et al., 2016); however, sudden events such as job loss or escalating family tensions may increase the risk of isolation (Holdsworth and McCabe, 2018; Werner et al., 2009). In addition, stigma and social invisibility may reinforce experiences of loneliness and foster strategies of normalization or concealment of symptoms (Clemerson et al., 2014; Lockeridge and Simpson, 2013; Tolhurst et al., 2014).

The literature highlights a high prevalence of unmet needs across several domains of everyday life, including daytime and occupational activities, social participation, intimate relationships, communication, memory, mobility, psychological distress, and information needs (Bakker et al., 2014). Among these, certain challenges appear particularly salient in YOD, as they are closely intertwined with key adult life trajectories, such as maintaining physical activity, sustaining employment, parenting, and managing financial responsibilities (Ottoboni et al., 2021; Sansoni et al., 2016).

Alongside the description of challenges and needs, it is essential to further explore the experience of dementia from the perspective of those who live with it (Robinson et al., 2012). Early work in this area has been strongly influenced by person-centered approaches, which have played a crucial role in shifting attention away from purely biomedical models and toward the recognition of personhood, relationships, and communication in dementia care (Kitwood., 1997; Robinson et al., 2005; Sakamoto et al., 2017). However, this framework has been critically revisited, with scholars highlighting its conceptual ambiguity and its tendency, in some instances, to privilege individual autonomy while underestimating the fundamentally relational nature of care (Brooker, 2003; Mitchell and Agnelli, 2015; Nolan et al., 2004). In response, more recent theoretical developments have foregrounded relational and relationship-centered perspectives, emphasizing that care is inherently embedded within networks of interdependent relationships involving people living with dementia, caregivers, professionals, and broader social contexts. Within this view, relationships are not merely contextual but constitutive of care itself, shaped by reciprocity, emotional engagement, and mutual influence (Beach et al., 2006; De Witt and Fortune, 2019; Nolan et al., 2004). This shift aligns with broader relational theories that conceptualize human experience as fundamentally interdependent and continuously shaped through social interaction (Jordan, 2004; Nedelsky, 2012). At the same time, there has been increasing recognition that the experience of dementia is not only internally lived but also actively negotiated through communication and interaction. Participatory and co-constructive approaches have highlighted the active role of people living with dementia in shaping meaning and contributing to knowledge production, challenging earlier deficit-based assumptions and the notion of dementia as a “loss of self” (Harris and Keady, 2009; Keady et al., 2007; Wilkinson Heather, 2002). These perspectives foreground processes of identity negotiation, adaptation, and relational meaning-making as central to the experience of living with dementia. Taken together, these developments support a shift toward understanding dementia as a relational and communicative phenomenon, in which experiences are continuously constructed and negotiated within everyday interactions.

In parallel, qualitative research in dementia has expanded to include a wide range of methodological approaches. Alongside interview-based designs, creative and participatory methods-such as drawing, storytelling, poetry, and theater-have been increasingly employed to support alternative modes of expression and inclusion (Kontos and Martin, 2013; Zeilig et al., 2014). Co-produced research has also gained prominence, involving people living with dementia as active contributors to the research process and challenging traditional researcher-participant hierarchies (James and Buffel, 2023; Littlechild et al., 2015). These developments reflect an increasing diversification of qualitative approaches, each offering access to different dimensions of experience.

Despite this methodological expansion, qualitative research has been predominantly conducted through semi-structured interviews (Busted et al., 2020; Greenwood and Smith, 2016; O'Malley et al., 2019; Pipon-Young et al., 2012; Rabanal et al., 2018; Roach et al., 2016; Robinson et al., 2012). While valuable, such approaches rely on researcher-defined questions and interactional structures, which may shape participants' accounts and orient responses toward anticipated expectations or normative frameworks (Kusenbach, 2003; Thomas, 2006).

In response, there has been increasing interest in context-sensitive and interactional methods, including ethnographic and mobile approaches such as go-along or walking interviews. These approaches generate data within everyday environments, capturing how experiences are shaped through ongoing engagement with social and physical contexts and enabling access to embodied, situated, and relational forms of knowledge (Bartlett et al., 2023; Carpiano, 2009; Kusenbach, 2003; Ward et al., 2018, 2022). In dementia research, they have been shown to reduce cognitive demands, support participation, and shift interactional dynamics by allowing participants to guide the encounter (Brannelly and Bartlett, 2020; Howard and Thomas-Hughes, 2021; Keady et al., 1995, 2007, 2017).

Within this methodological landscape, spontaneous peer-to-peer conversations represent a distinct approach to data generation, characterized by a reduced degree of researcher-imposed structure. Unlike interviews or other elicited methods, these interactions are not organized around externally defined questions but emerge from participants' own orientations, priorities, and relational dynamics.

This does not imply that such data are inherently more “authentic” than those generated through other qualitative approaches. Rather, they provide access to a different layer of experience: the situated, interactional production of meaning as it unfolds in real time. By minimizing researcher intervention, these interactions allow participants to shape the interaction according to their own concerns and priorities, while reducing the extent to which contributions are oriented toward externally imposed questions or anticipated expectations. In this way, they provide access to the situated, interactional production of meaning as it unfolds in real time.

Against this backdrop, the present study adopts an approach based on the analysis of spontaneous conversations among people living with YOD. By focusing on naturally occurring interactions, the study aims to explore how experiences, needs, and coping processes are articulated and negotiated in real time, foregrounding participants' own orientations without imposing externally defined structures.

## 2 Methods

Ethical approval was obtained from the University of Bologna Ethics Committees [Ref: 0313536] in September 2024.

All study participants provided written informed consent, confidentiality and the participant's right to withdraw at any time.

### 2.1 Study design

We conducted a qualitative exploratory study, drawing on naturally occurring group conversations among people with YOD. The research design was based on a general inductive approach that allowed themes to be generated directly from the data without constraints imposed by structured methodologies or predefined hypotheses (Thomas, 2006). The choice of this methodology is consistent with the aim and the exploratory nature of the study, which envisages the active involvement of people with YOD in the research process (Fudge et al., 2007; Littlechild et al., 2015), recognizing them as key experts on the topic.

### 2.2 Participants and recruitment

Participants were people with a diagnosis of YOD (diagnosis before the age of 65 years) of any type, who were aware of their diagnosis and did not present behavioral symptoms that could interfere with group participation. Participants were recruited through convenience sampling through a Meeting Center specifically dedicated to people with YOD in Italy. Initially, members of the Meeting Center staff presented information about the study and invited them to participate. Subsequently, researcher took part in weekly meetings. All people attending the Meeting Center during the data collection period agreed to take part in the study. Recruitment was stopped when no new themes emerged during the analysis of three consecutive group conversation sessions, indicating that theoretical saturation had been reached (Saunders et al., 2018). The participants were thanked for their involvement and the findings were shared with them in a feedback session.

### 2.3 Data collection

Data collection spanned a period of several months, between October 2024 and February 2025, allowing for multiple sessions and the emergence of diverse and heterogeneous narratives. Data were collected through audio-recordings of face-to-face conversations among participants. The activities at the Meeting Center involve the presence of two experienced psychologists and take place once a week. The activity program is divided into four main phases: free dialogue, coffee break, cognitive stimulation, and aerobic activity based on the dance movement. However, the focus of the study is on the part of the activity devoted to free dialogue, i.e., a space dedicated to informal conversation around a table that allows group participants to express themselves, share experiences, ask for advice and converse freely without leading questions. The conversations recorded were naturalistic group interactions: no topic or prompt was proposed in advance, and researcher did not intervene in the dialogues. The role of the psychologists was limited to occasional minimal facilitation when needed (e.g., encouraging quieter participants to contribute, ensuring turn-taking, or clarifying references to maintain mutual understanding). The recordings, each lasting approximately 100 min, were conducted by the main author (M.Q.), who was external to the service of the Meeting Center. None of the participants knew the researcher beforehand. At the beginning of the study, socio-demographic characteristics about participants (e.g., age, gender, level of education) and condition (e.g., time and type of diagnosis) were collected.

### 2.4 Data analysis

All group conversations were recorded in audio format and subsequently transcribed verbatim for analysis by the lead author (M.Q.). Each participant was assigned a pseudonym to ensure anonymity and protection of personal data. All data were analyzed in Italian, the original language of the participants. Only the excerpts included in the manuscript were translated into English for reporting purposes, with careful attention to preserving meaning and interactional features. The data were analyzed independently by two researchers (M.Q. and L.F.) using the general inductive approach (Thomas, 2006). The analysis began with a repeated reading of the transcripts to familiarize themselves with the contents. Each researcher manually identified significant segments related to the experience of living with dementia, using *in vivo* coding, and assigned labels directly from the words used by the participants. The related labels were grouped into categories, subsequently organized into broader themes, again by each researcher. The iterative coding process led to the construction of a shared thematic structure, which was progressively developed. Differences in coding and interpretation were systematically examined, and consensus was reached through dialogue between the two researchers (M.Q. and L.F.) for each transcript. At the end of the process, the results of the analysis were discussed with a third researcher (R. C.), reaching a consensus on the consistency of the themes that emerged and resolving any discrepancies (Hickey and Kipping, 1996). The findings were then discussed with the wider research team to ensure coherence and conceptual clarity. Participants were involved in the interpretive phase through a feedback session, during which preliminary themes were presented and discussed. They were invited to reflect on their alignment with lived experience and to suggest refinements. Their contributions informed both the interpretation of findings and the wording of themes. Finally, the results were shared with participants during a Meeting Center group session, where themes were further discussed and refined. This process can be understood as a form of participant validation, contributing to the credibility and trustworthiness of the findings (Birt et al., 2016; Lincoln and Guba, 1985).

## 3 Findings

### 3.1 Sociodemographic characteristics

Fourteen spontaneous group conversations were collected, involving 11 individuals with a formal diagnosis of YOD. Participants ranged in age from 55 to 68 years (*M* = 62.18), and all were of Italian nationality. The majority were male (*n* = 7; 63.6%). The most frequently reported level of education was lower secondary school (*n* = 5; 45.5%). Regarding marital status, most participants were married (*n* = 8; 72.7%) and lived with their spouse or partner (*n* = 5; 45.5%). At the time of data collection, the majority were not employed (*n* = 9; 81.8%). The most common diagnosis was Alzheimer's disease (n = 6; 54.5%), with a mean time since diagnosis of 21.7 months, ranging from 2.6 months to 6 years. Sociodemographic and clinical characteristics are summarized in Table 1.

**Table 1:** Sociodemographic and clinical characteristics of participants.

Characteristics	Category	Frequency (n)	Percent (%)
Gender	Male	7	63,6%
	Female	4	36,4%
Educational level	Low secondary education	5	45,5%
	High school diploma	4	36,4%
	Postgraduate degree	1	9,1%
	N/A	1	9,1%
Marital status	Married	8	72,7%
	Divorced	3	27,3%
Living status	Lives with spouse/partner	5	45,5%
	Lives with family (children present)	3	27,3%
	Lives alone	3	27,3%
Employment status	Not employed	9	81,8%
	Employed	2	18,2%
Type of diagnosis	Alzheimer's disease	6	54,5%
	Not specified	3	27,3%
	Lewy body dementia	1	9,1%
	Vascular dementia	1	9,1%

N/A: not available data.

### 3.2 Qualitative results

The thematic analysis of spontaneous group conversations with people with young-onset dementia identified four interconnected themes that illustrate how participants make sense of and respond to their condition.

After developing the preliminary findings, these were presented back to participants, who had the opportunity to collectively reflect on the themes that had emerged. At this stage, participants largely confirmed the results but also suggested modifications and clarifications—for instance, in the choice of words or in the articulation of nuances—which were incorporated into the final version. In this way, the results presented here do not solely reflect the researchers' analysis but were co-constructed together with the participants, respecting their interpretations, linguistic preferences, and experiential perspectives.

Table 2 provides a schematic representation of the main themes and related subthemes, which will be further elaborated in the following sections.

**Table 2:** Themes and sub-themes as identified by participants.

Themes	Sub-themes
1. Living with Alzheimer's: between awareness and “broad shoulders”	Naming what happens
	Emotional expression and confronting loss
	“Not being crushed”
	Finding new meaning in a changing life
2. Significant relationships: between closeness, misunderstanding, and new balances	Couple relationships
	Relationships with children
	Family relationships
	“We are the Alzheimer's group”
3. The struggle to be believed: between invisible illness and judging gazes	Social recognition of an invisible condition
	Unfair behaviors and stigmatizing attitudes
4. Support that makes a difference: between sources and ideas for living better	Family support
	Professional support
	Being helped in the right way

#### 3.2.1 THEME 1: Living with Alzheimer's: between awareness and “broad shoulders”

##### 3.2.1.1 Naming what happens

One of the most difficult and transformative stages in living with the disease is naming the symptoms, recognizing that the cognitive and emotional changes experienced are not temporary, but rather part of a progressive condition. It is often a stage marked by denial, disorientation, fear, and a sense of inadequacy:

“*I felt very small when I received the diagnosis.”*

“*The horizon changes after the diagnosis. Self-esteem immediately decreases, there is fear because you do not know what to expect. There is a shock. It is also a shock for loved ones, because it is difficult to make them understand.”*

The initial temptation to deny the condition was common, as described by those who struggled to recognize themselves in the diagnosis or to share it with others:

“*The temptation to refuse this thing… I had it when I went to the doctor. I said: ‘No, it's not true… what am I doing here, what do I have to do with them?”*

Many participants reported the initial attempt to conceal symptoms, often experienced as subtle signals that were difficult to acknowledge:

“*At the beginning you try to sweep the dust under the doormat… Sometimes you ask yourself: ‘Why me?”'*

Over time, however, greater awareness of the illness and its impact on everyday life developed:

“*This illness has changed our lives… practically, emotionally… but this is also our strength.”*

Other participants described episodes of disorientation and loss of control over their daily lives, in which the narrowing of autobiographical memory became a breaking point that forced them to recognize the diagnosis:

“*I tried to remember… my memories go back20 years, from then until now I cannot remember anything.”*

##### 3.2.1.2 Emotional expression and confronting loss

The conversations revealed the emotional intensity of living with YOD, with recurring expressions of anger, sadness, fear, and frustration:

“*I am afraid, it is incredible, I say to myself ‘Where am I?'… not only do I fail to recognize places, but I also cannot remember which road I took.”*

Participants emphasized that the illness did not only affect memory or cognitive functions, but profoundly undermined their sense of security, autonomy, and continuity:

“*The difficulty I experience when there are too many people is debilitating. In the street, for example when going to the stadium and there is a large crowd, I feel uncomfortable and need to identify a point of reference.”*

“*One feels ashamed to admit that you can no longer do what previously was normal.”*

For many, emotions were deeply intertwined with the anticipation of future losses. This dynamic emerged through an oscillating pattern, characterized by an alternation between periods of apparent stability and phases of deterioration:

“*It feels like a board game… forward and backward, forward and backward.”*

##### 3.2.1.3 “Not being crushed”

Participants shared a variety of everyday practical strategies for addressing cognitive difficulties, and coping was described as a daily act of “not being crushed.” Some participants reported the use of compensatory techniques to deal with memory gaps and disorientation, such as relying on personal associations or landmarks:

“*In my memories I go by associations… the center is near the barracks, so I got off when I saw the barracks, otherwise I would have gone past.”*

Others adopted communication strategies to avoid losing track of what they wanted to say:

“*I speak quickly, otherwise… I lose the words.”*

Engagement in stimulating activities, such as reading, crosswords, or attending cultural lectures, was considered useful not only for keeping cognitive functions active:

“*I study a lot… I do it precisely because of the Alzheimer's, I try to remember.”*

Several participants also emphasized the importance of alternating between activity and rest, recognizing their limits while still sustaining participation:

“*I keep myself busy, in my own small way… then I get tired, I have to go to sleep and afterwards I start again reading or doing exercises to keep my mind trained.”*

##### 3.2.1.4 Finding new meaning in a changing life

For many participants, living with the condition was gradually integrated into their personal trajectory, becoming part of their biography without defining it entirely. A recurring feeling was: “We're not finished.”

Taking part in group meetings, discussing cultural topics, or simply “being together” with peers provided opportunities for connection and belonging:

“*I enjoy going out together because we meet up! Having an appointment to be together is good for me.”*

Engagement in cultural and learning projects was experienced as a way to feel competent and part of a group, contributing actively and keeping the mind alive in a context that was often light-hearted and playful.

“*I had prepared myself for the cultural meeting because I wanted to know beforehand and arrive prepared… I am interested, I have a passion… when I don't know something or don't remember it, I write it down so I can look it up again.”*

“*I come willingly… even just to see if I can beat them!”*

#### 3.2.2 THEME 2: Significant relationships: between closeness, misunderstanding, and new balances

##### 3.2.2.1 Couple relationships

Participants recounted how, at times, the partner's reactions to difficulties, though unintentional, could exacerbate anxiety, underscoring the importance of more empathic and attuned communication. These episodes illustrated the delicate balance between reassurance and pressure, and the need for reciprocal attunement within the couple:

“*When I am a bit out of breath I say: ‘I can't see well'… my wife replies: ‘What do you mean you can't see? What do you mean you can't see?'… and then I panic, and it gets worse.”*

Participants also described how, in everyday life, the partner's words could have a profound impact:

“*She made me really angry yesterday… she said a word she shouldn't have. She called me ‘stupid'… and with malice. They don't understand.”*

“*I dwell on everything she says to me, her words hurt me.”*

In some cases, participants described the difficulty of expressing their distress in order not to burden their partner. This self-censorship reflected a dynamic of mutual protection, in which love and care intertwined with silence in an attempt not to add further pain.

“*I try not to make it weigh too much on her… she already does a lot. Sometimes I prefer to pretend nothing is wrong.”*

At the same time, participants acknowledged the heavy load borne by their partners and, implicitly, the challenge of maintaining full symmetry within the relationship:

“*Our wives, husbands… they have quite a lot to do.”*

Despite these relational tensions, *it seems that* the partner's presence was often experienced as a source of reassurance and protection. This kind of “invisible support,” which safeguarded without exposing vulnerability, was particularly appreciated as it did not call into question one's dignity or autonomy:

“*When I go out with her [my partner] I feel calmer… she knows where we are going, she guides me even if she doesn't show it.”*

At the same time, however, there was also a desire not to be defined solely as someone in need of help, but as a partner with whom to share words, emotions, and reciprocity:

“*She reminds me of everything, I rely on her… but there are moments when I would just like to talk, not ask for help.”*

##### 3.2.2.2 Relationships with children

The conversations revealed that the illness profoundly reshaped the parent–child relationship, even in cases where children had not actually been brought into the world.

One participant, in fact, reported having consciously decided not to have children for fear of passing on the illness. This choice was strongly tied to the perception of a hereditary component of dementia, experienced as a threat to future generations:

“*I did not want to have children, because in my family my grandfather, uncle, and aunt on my mother's side all had Alzheimer's, so I preferred not to have a family for this reason.”*

Where children were already present, the relationship was described as loaded with complex emotional tensions. Some participants spoke with bitterness about the difficulty of communicating with their child, who often did not trust them or became angry at forgetfulness or difficulties linked to the illness. It was not only the illness that created distance, but also the struggle to be recognized for who they were in the present, within a fragile balance between continuity and change:

“*The problem is that my son doesn't trust me […] he gets angry with me too.”*

“*My son doesn't understand that I have changed, that I am no longer the same as before… if I don't drive anymore and I don't cycle anymore, there must be a reason, but he doesn't understand.”*

In this context, the tone of voice and the emotional attitude of the family member became central. The emotions of others could be particularly painful or destabilizing. A profound desire emerged to be respected, understood, and accompanied, rather than corrected or monitored. Even in moments of tension, the need for affective communication remained central:

“*I always tell my son: ‘I've always done everything, you must understand that now something is not right, no? So calm down and don't shout, because I don't like an authoritarian voice. I never liked it, and now even less. It bothers me even more than before.”'*

“*The other time my son said to me: ‘Are you stupid?' and I slapped him twice. I told him: ‘Be careful how you speak. I never slapped you before, but now I did, because I don't like the way you talk to me […]'. Now he has learnt. And I always tell him: ‘Because the more I go to work, the less I lose, you see?”'*

##### 3.2.2.3 Family relationships

Participants recounted experiences in which relatives—especially those less involved in daily care—appeared harshly judgmental, dismissive, or unable to grasp the changes brought by dementia:

“*My uncle sees me with the trolley and says: ‘Come on, don't make fun of me.' Sometimes I fall… but I can't say it, because once I told him: ‘Uncle, look, I fall,' and he replied: ‘Oh, come on, someone like you, really…”'*

Family members often continued to project a static image of the person—the one they “*used to be”*—fuelling distance and disconnection:

“*They see you, they take a picture, they hang it on the wall and that's it… but in life, we change.”*

Yet some participants also stressed the presence of deeply meaningful family ties, especially with grandchildren, who offered moments of connection and motivation:

“*I have three grandchildren who give me strength. They understand more than the adults.”*

They did not necessarily grasp the complexity of the diagnosis but reinforced continuity and the desire to remain active in one's family role:

“*They say: ‘Grandfather, we know the way, don't worry'… they are happy that grandad takes them to the rides.”*

##### 3.2.2.4 “We are the Alzheimer's group”

A recurring theme in participants' accounts was the central role of the peer group as a safe, welcoming, and strongly identity-shaping space. Self-identifying as “*we are the group with Alzheimer's”* was not expressed with shame, but rather with a sense of pride and recognition.

From the very first meetings, the sense of community assumed a shared symbolic and emotional meaning. The choice of the group's name reflected this collective sentiment, through a metaphor encapsulating the daily effort required to maintain balance and vitality:

“*The (name of the group) because it always has to keep working… otherwise it falls.”*

“*I remember when we discussed choosing the name, we wanted something that was linked to the difficulties of our condition, the reason why we are here in this group.”*

The group became a space where the diagnosis could be openly stated and shared without fear of judgement:

“*I always say that we are the Alzheimer's group.”*

Participants emphasized the contrast between the outside world, perceived as judgemental or ill-informed, and the group, experienced as a protected environment:

“*When I am here, I feel as if I were at home… when we talk, you know what I have, I know what he has. So, we are at ease among ourselves, whereas outside… it's a jungle.”*

The group fostered meaningful bonds that went beyond the diagnosis, transforming initial reluctance into trust, connection, and openness:

“*I didn't want to come here, because I thought it wasn't useful for me, but actually it is.”*

“*But it's nice, when you go back home, not to shut yourself in, but to say ‘look, we've done something good… and if the word you want doesn't come to you in 3 seconds, who cares.”'*

“*I hope we get along well and become dear friends, because we have to be friends, right? Not only here, but also outside.”*

The group represented a space where even the most difficult emotions could be acknowledged. Peer exchange allowed fears and vulnerabilities to be addressed collectively, transforming subjective experience into shared narrative:

“*We are a stronghold that nothing can break.”*

“*You can share… we are a group!… We are a bit like a family too.”*

Within the group, each member perceived themselves as being on the same level, regardless of personal differences or stage of illness. This sense of equality fostered dignity and mutual recognition, counteracting the feelings of inferiority and marginalization often associated with the diagnosis:

“*It's nice to be here because at this table, we are all equal.”*

“*We are all in the same boat, and it's a good boat.”*

“*I feel safer, free to say silly things… I feel free, I feel better. You can be free to say what you want, to joke, to make fun. A different atmosphere is created when working in a group.”*

The group is described as a positive, effective, and transformative example:

“*Being with others is already half a cure.”*

“*The group works because of the exercises we do, because we work as a team, and because we are all young.”*

#### 3.2.3 THEME 3: The struggle to be believed: between invisible illness and judging gazes

##### 3.2.3.1 Social recognition of an invisible condition

The fact of appearing “normal” fuelled doubts and suspicions, forcing participants to continually justify their condition:

“*When I talk about Alzheimer's, everyone says to me: ‘But how can you have it? You're so active.' Once someone told me: ‘No, you can't have Alzheimer's… it's not possible.”'*

“*My uncle says I'm making fun of him… he sees me with the trolley and says: ‘Come on, don't make fun of me.”'*

Social recognition was thus refused or delayed, and the diagnosis had to be constantly defended by those living with it. In this context, many reported adapting themselves to others in order to avoid misunderstanding:

“*It's us who adapt, because they don't understand.”*

“*The hardest thing for us is to camouflage ourselves. But if we want to survive, we have to camouflage ourselves.”*

Yet this adaptation came at a cost: it often resulted in silence, emotional fatigue, and the renunciation of full self-expression. Participants spoke of a form of silent struggle:

“*We fight in silence, but others do not notice. And where is our inner pain recognized?”*

“*This is not a society that forgives diversity: being ill, being considered strange.”*

##### 3.2.3.2 Unfair behaviors and stigmatizing attitudes

Many described the feeling of being judged through the gaze of others – a gaze that transformed everyday mishaps into signs of incompetence or weakness, rather than symptoms of a neurological condition:

“*They look at me differently compared to 4 or 5 years ago. Now they don't just look at me: they scrutinize me, as if to say, ‘Sooner or later you'll make a mistake.”'*

Unjust behaviors also became evident in everyday life, often in public settings:

“*I went to the hospital bar… someone cuts in front of you, they don't care… they don't respect other people's time.”*

“*Even just in the supermarket queue, if I take a little longer people get angry and scold me. Then I get nervous too, and I reply: ‘Sorry, I'm doing what I can.”'*

Similar situations occurred in more formal contexts, such as meetings or cultural events, where speakers did not allow them to intervene or share their experiences:

“*The feeling I had was that of being a fish out of water. They isolated us. I asked questions and they silenced me. They made us feel uncomfortable.”*

“*We all kept quiet, only the professors spoke. I thought we would be able to talk, but when they saw us… they only spoke among themselves. They weren't even interested in who we were, they were just focused on their own group.”*

Participants described everyday experiences of vulnerability related to the risk of being deceived or taken advantage of due to memory difficulties:

“*People take advantage of you because you don't remember, so they make you pay for two coffees instead of one. I don't go to the bar alone anymore, because I know there's always someone ready to take advantage.”*

#### 3.2.4 THEME 4: Support that makes a difference: between sources and ideas for living better

##### 3.2.4.1 Family support

A recurring theme was the request that relatives give them time, space, and the opportunity to act independently, even if this meant tolerating mistakes or slowness:

“*I don't want others to think I can't manage… I need to do things at my own pace, and then I get there.”*

Some participants described feeling judged or obstructed even when attempting to maintain certain forms of autonomy:

“*They say to me, ‘Leave it, don't drive anymore'… but I haven't had any accidents, just because I forget things?”*

The most effective family support was that which did not impose but accompanied. As participants reflected, what made the difference was not only practical help, but the quality of the relationship, based on trust, listening, and respect for personal timing:

“*It's enough if they tell me: ‘ok, take your time'… and I feel better. I don't want them to do everything for me.”*

While acknowledging difficulties and moments of tension, they also expressed gratitude and understanding for their relatives' commitment:

“*If a family member gets angry from time to time, you can forgive it, because they do so much for us.”*

##### 3.2.4.2 Professional support

They recognized the value of competent practitioners but criticized the tendency of services to propose standardized solutions that failed to capture the nuances of living experience:

“*Because those of you who work with people like us — doctors, assistants and all those who support us — you have the books on Alzheimer's, right? […] But in reality I've seen that there is a common basis, yet each of us has completely different characteristics. […] That's why it's difficult to find the right way: because we are all very different from one another.”*

When professionals were able to build authentic relationships and attune themselves to people's living experiences, the support received could become a source of relief and trust:

“*It may be that I am not able to explain what is happening to us… Now I don't think about it so much, but before I did. Now I've almost let it go… In short, it's clear that you need someone beside you, eh… For some time now I've been less afraid.”*

Within this framework, professional support emerged as a crucial resource, not only for clinical assistance but also as a relational tool within the family:

“*Afterwards, when the psychologist came to our home and explained everything clearly to my son, he changed. Before, he didn't understand.”*

The presence of psychologists and facilitators within the dedicated group was particularly valued:

“*We have found a wonderful staff… when we laugh, it's a beautiful thing.”*

##### 3.2.4.3 Being helped in the right way

Participants emphasized that improving their situation would require greater training for family members, often described as affectionate but unprepared to cope with the transformations brought about by the illness:

“*Families need to be educated because they don't understand what Alzheimer's is. They don't know.”*

The lack of specific knowledge was perceived as a source of misunderstanding and frustration:

“*The problem is that we already have the condition, so there's no point in them getting angry… before saying something they should count to three, relax a little, and then speak… but instead they don't.”*

Participants proposed a concrete strategy: the presence of an expert mediator, considered useful in helping families to better understand the condition when direct words were not enough:

“*I told them: ‘When I go to the psychologist, come with me so she can explain what you have to do.”'*

Participants stressed that the emotional climate directly influenced cognitive performance. Speaking calmly and gently was experienced as a strategy of facilitation and respect:

“*I always say: I am the same as before, just with less memory… Without raising your voice, calmly, I can do everything… I know I struggle to understand and that I get lost, but if you are a bit gentler I get lost less.”*

Exclusion experienced during public events and cultural initiatives was interpreted as the result of a lack of sensitivity and preparation toward people with YOD. Participants suggested a preventive approach:

“*Maybe they should be made more aware… so that when they are faced with a group of people with Alzheimer's, they will know how to behave better.”*

The idea of providing a clear introduction before events was proposed to avoid embarrassment, confusion, and a sense of exclusion. According to participants, both the public and organizers need training to make spaces more accessible and welcoming:

“*Perhaps next time it would be useful to organize activities designed especially for us and to know who we are without us having to explain.”*

Participants also strongly expressed the desire to be actively involved in the design of spaces, activities, and centers tailored to their age and needs:

“*We should open more centers dedicated to us. Like a compass, because we get lost.”*

“*We should tell those who don't know what this illness really is… It is important to say how we truly live.”*

## 4 Discussion

This study was designed to explore what people living with YOD have to say about their experience of the condition, moving beyond the methodological boundaries of structured interviews (Potter and Hepburn, 2005; Saigh, 1992). Rather than aiming to identify strictly novel thematic content, the original contribution of this study lies in the way the living experience of YOD is explored, made visible, and co-constructed. Through the analysis of naturally occurring peer-to-peer conversations, the research foregrounds participants' direct voices and captures relational, linguistic, and identity processes as they unfold within natural peer interactions.

In line with the literature, the findings show that the communication of the diagnosis represents an existential turning point, marking a biographical rupture and a sudden redefinition of one's idea of the future (Birt et al., 2017; Buckman, 1984; O'Malley et al., 2019). Giving a name to what is happening emerges as a gradual process that takes time and often entails a complex confrontation with changes in the self. This process unfolds within a strongly value-laden cultural context. Social representations of dementia are predominantly centered on decline, loss and dependency. These representations shape expectations, living experiences and interpretations from the very early stages of diagnosis (Fazio, 1996). Our data further suggest that, over time, the diagnosis tends to be progressively integrated into one's personal life story, allowing for a reorganization of the meaning attributed to the experience.

Within this process, language plays a central role. Consistent with previous studies, the term *dementia* is associated with highly stigmatizing representations and an image of global incompetence (Braudy Harris and Sterin, 1999; Langdon et al., 2007). The findings align with this body of evidence, showing that the preference for the term *Alzheimer's* does not reflect a denial of the condition, but rather an active and identity-affirming choice. Participants consciously adopt the term *Alzheimer's* even in the presence of heterogeneous clinical forms, as it is perceived as referring to a disease in an organic and neurological sense, rather than as a global label incorporating moral, social and stigmatizing attributes. This choice can be interpreted as a form of symbolic resistance to stigma, aimed at preserving dignity, continuity of the self and social legitimacy. In this sense, language does not merely describe the illness, but actively contributes to organizing living experience and shaping the person's social positioning (Goffman, 1974). In contrast with studies reporting that clinicians and family members tend to avoid the term *Alzheimer's* in order to reduce its emotional impact (Antoine and Pasquier, 2013; Boise and Connell, 2005; Langdon et al., 2007), our data suggest a different perspective. For people directly affected, explicit naming may instead serve a function of clarity and recognition. In continuity with earlier contributions (Bender and Cheston, 1997; Clare, 2003; Keady et al., 1995), the findings thus highlight the importance of actively involving people with a diagnosis in the terminological choices that concern them, acknowledging language as a central device in the construction of identity and living experience (Goffman, 1963).

Alongside this, participants tend to avoid references to *resilience*, preferring the expression “*having broad shoulders,”* which conveys the everyday weight of the illness and the ongoing effort required to carry it. This formulation can be interpreted as a spontaneous form of reframing, implicitly rejecting a heroic or idealized reading of the experience and instead restoring the dimension of continuous, unresolved effort that accompanies living with the condition (Benson et al., 1992; Fazio, 1996).

The findings further show that YOD is accompanied by persistent emotional responses and an ongoing process of anticipatory grief that does not end in the period immediately following diagnosis, but continues to shape living with the condition over time (Greenwood and Smith, 2016; Rabanal et al., 2018; Waddington et al., 2024). Social interactions appear to play a central role in modulating these experiences: in contexts marked by limited understanding and lack of recognition, emotional distress tends to intensify, translating into experiences of exclusion and loneliness (Oliveira et al., 2023). Our data do not contradict the existence of more favorable adaptation trajectories over time (Johannessen et al., 2019; Molvik et al., 2024; Thorsen et al., 2020), but rather suggest that suffering is more likely to persist when social recognition is lacking.

Living with dementia entails the mobilization of everyday strategies. These include compensatory techniques, communicative adaptations, careful management of energy, and engagement in meaningful activities. Coping thus emerges as an active endeavor aimed at preserving agency, a sense of competence, and balance in everyday life. In line with the literature, the diagnosis is not interpreted as the end of one's life, but rather as a condition within which it remains possible to continue participating, maintaining relationships, and being included in society (Moe et al., 2021; Persson et al., 2023; Pipon-Young et al., 2012; Thorsen et al., 2020). The data also suggest a shift in focus from individual attitudes to the relational processes that make such an orientation possible. Shared activities and peer relationships appear to foster continuity of the self and the construction of meaning (Fazio, 1996).

With regard to couple relationships, the findings confirm the asymmetrical reconfiguration widely described in the literature (Ablitt et al., 2009; Hellström et al., 2007; Keady et al., 1995), while adding fine-grained insights at the micro-interactional level. In particular, language, tone, and communicative styles used by the partner appear to modulate anxiety, sense of competence, and emotional security in an immediate way. Moreover, the use of strategies of self-censorship of distress emerges as an active relational choice, aimed at protecting the partner and preserving the balance of the relationship. Finally, couple intimacy appears to follow non-linear trajectories, oscillating between functional reduction and reinforcement as an affective and identity-related resource, thereby extending previous evidence on the maintenance of couplehood (Evans and Lee, 2014; Hellström et al., 2007).

With regard to the parent–child relationship, the findings are consistent with the literature describing its vulnerability in YOD (Gelman and Rhames, 2018; Millenaar et al., 2014; Svanberg et al., 2011), while introducing the direct perspective of parents living with dementia. Judgement and infantilisation emerge as experiences that are particularly threatening to dignity and adult identity, with potential repercussions for major biographical decisions, including the decision to forgo parenthood (Fahy et al., 2025; Quaid et al., 2010; Werner et al., 2009).

Extended family relationships appear ambivalent, oscillating between support and misunderstanding, particularly in the presence of limited awareness of the condition (Langdon et al., 2007; Lyman, 1993; Oliveira et al., 2023). In line with critiques of medicalisation, what is not recognized as living experience risks being interpreted as deviance or incompetence (Fazio, 1996; Lyman, 1993). Alongside these dynamics, some intergenerational bonds, particularly with grandchildren, emerge as symbolic and affective resources (Venters and Jones, 2021).

Peer groups are confirmed as a relevant resource (Greenwood and Smith, 2016; Rabanal et al., 2018), functioning as spaces for the collective reframing of experience, in which the illness is normalized and integrated into everyday life (Fazio, 1996; Kitwood., 1997). Group belonging fosters equality, freedom of expression and opportunities for friendship, in contrast with experiences of judgement in the wider social context (Craig and Strivens, 2016; Keyes et al., 2016; Ward et al., 2012).

Because the signs of the condition are not immediately visible and age contradicts the dominant social imaginary, people with YOD often find themselves having to legitimize their diagnosis, turning everyday life into a continuous effort of explanation and self-defense (Greenwood and Smith, 2016; Oliveira et al., 2023). Masking strategies may allow only an apparent form of social participation, but at the cost of substantial emotional effort, in line with processes of identity management and social discredit described in the literature (Goffman, 1963).

Our findings align with recent evidence showing that people living with dementia not only experience stigma and social judgement, but are also exposed to systemic forms of epistemic injustice (Fricker, 2007), in which their capacity to express and legitimize their own experience is devalued or denied (Calabrese et al., 2026). The minimization of everyday difficulties in public or institutional contexts contributes to experiences of stress, anger and humiliation (Bhatt et al., 2023). It can also be understood as a form of epistemic exclusion that reinforces stereotypes of incompetence and marginality (Halonen et al., 2025). In partial contrast with studies highlighting the protective role of more inclusive environments (Johannessen et al., 2019; Thorsen et al., 2020), the data suggest that, in the absence of adequate epistemic recognition, everyday micro-injustices tend to persist, fostering social withdrawal and limitations in autonomy.

The family emerges as a central resource, yet it may also become a source of suffering when support takes hyper-protective or devaluing forms, thereby hindering genuinely shared care (Chemali et al., 2012; Podell and Torres, 2011; Sampson et al., 2004). When read through a reframing lens, this dynamic highlights how the interpretative frames adopted by family members shape relational responses, directly affecting the recognition of residual competencies and the adult role of the person living with dementia (Fazio, 1996; Lyman, 1993; Pesut, 1991). Participants emphasize the importance of support grounded in respect, listening and trust in individual rhythms, capable of preserving autonomy, dignity and reciprocity, and clearly distinguishing *being alongside* from *doing things in place of* the person.

Our findings are consistent with the literature indicating that standardized and impersonal approaches within dementia services may hinder recognition of the person and weaken the caring relationship. In contrast, person-centered interventions promote trust and wellbeing (Edvardsson et al., 2008; McCormack and McCance, 2016). Within this framework, the value of professional support emerges when it takes on a mediating role between the person and the family, facilitating a more nuanced understanding of the condition and a revision of attitudes and expectations, in line with evidence on professionally mediated psychoeducational interventions (Gaugler et al., 2007; Mittelman et al., 2006). From the perspective of people with YOD, psychologists and facilitators are therefore perceived as key figures not only for practical management, but also for emotional containment and the creation of a climate of trust.

The findings show that participants tend to interpret social exclusion not as an inevitable outcome, but as a largely preventable and structural problem. This highlighting the need for training, awareness-raising and inclusive design interventions within public and cultural spaces, which are still often poorly adapted to the needs of people with YOD (Tolhurst et al., 2014). In line with a co-production approach, and going beyond a logic of mere consultation, participants describe themselves as competent interlocutors and call for more active involvement in the design of services and activities, in continuity with evidence that values experiential knowledge in the development of more appropriate and sustainable responses (Mayrhofer et al., 2020).

### 4.1 Strengths and limitations

This study has several strengths. First, the study used group conversations collected in a real-world setting. These conversations were not based on an interview guide and did not include researcher-introduced questions or prompts. This approach allowed us to directly observe processes of meaning-making, the use of humor, peer negotiation, and the construction of collective identity. These dimensions are difficult to capture with the same level of detail through individual interviews and therefore provide a complementary contribution, with a strong degree of ecological relevance. Second, participatory involvement-through the feedback of findings and the integration of clarifications and terminological preferences-strengthened interpretive credibility and foregrounded the first-person perspective of people with YOD as an epistemic resource. A further strength lies in the temporal richness of the dataset: data collection conducted over several months across 14 conversations supported the emergence of both recurring and heterogeneous narratives, enabling the observation of continuity and change in content over time. Finally, the Italian context represents an original contribution within a field still largely dominated by Anglo-Saxon and Northern European evidence, enhancing the cultural transferability of knowledge on the living experience of YOD.

With regard to limitations, participants were recruited from a single support context, potentially characterized by existing resources, facilitated access to services, and relatively active engagement. This may reduce the transferability of findings to people with limited or discontinuous access to services, or greater socio-familial vulnerability. Another limitation concerns the relatively small sample size; moreover, the inclusion criteria may have excluded individuals at more advanced stages or with more complex presentations, resulting in the possible under-representation of specific needs and challenges. The predominance of Alzheimer's disease diagnoses also limits the applicability of conclusions to other forms of YOD (e.g., FTD), which may involve different clinical and psychosocial trajectories. Similarly, certain sample characteristics (male predominance, Italian nationality, age range 55-68 years) may reduce generalisability to groups with different cultural and socioeconomic backgrounds.

As the data were collected and analyzed in Italian and only translated into English for reporting purposes, some linguistic and interactional nuances may not be fully captured in the translated excerpts. A further limitation relates to group-based conversational dynamics: even in the absence of guidance, peer interactions may encourage conformity, self-censorship, and selective disclosure, making some topics more readily shareable (e.g., belonging, collective identity) and others less likely to be explored (e.g., sexuality, depressive ideation, family conflict). Finally, the presence of the centre's psychologists and the care environment of the Meeting Center may have implicitly shaped communication styles and content (i.e., a setting effect).

In light of these limitations, future studies should replicate this conversational approach in more diverse contexts and with more heterogeneous samples in terms of sociodemographic and diagnostic profiles. In particular, the dimension of social vulnerability and the risk that others may take advantage of their difficulties-an original theme emerging in this study-warrants further investigation, given its potential relevance for the design and evaluation of community-based interventions and safeguarding strategies.

### 4.2 Conclusions and implications

This study adopted a data collection approach that enabled an in-depth understanding of the living experience of young-onset dementia (YOD) directly through participants' authentic voices, valuing first-person perspectives and meanings that emerged spontaneously. The findings have psychosocial and organizational implications. These implications are grounded in what participants describe as necessary to feel supported in their everyday lives.

At a psychosocial level, the findings highlight the importance of support that addresses identity, relationships, and participation in everyday life, alongside clinical aspects. Supporting continuity of identity, preventing infantilisation and stigma, and promoting more competent and collaborative family communication emerge as key priorities. These findings point to the need for approaches that sustain the person's social role, meaningful relationships, and active participation.

At the level of service organization, the findings highlight the importance of interventions that respond to the relational and communicative needs of people living with YOD. Participants emphasized the need for greater support and training for family members, as limited understanding of the condition was associated with misunderstanding and distress. The involvement of professionals as mediators was identified as a useful strategy to facilitate communication and support more adaptive relational dynamics. The findings also point to the importance of communicative environments characterized by calmness and respect, as these were described as directly influencing participants' ability to engage in everyday interactions. At a broader level, the need for increased awareness and preparedness in public and community settings emerged, together with the importance of age-appropriate, dedicated services. Taken together, these findings suggest the need for service models that integrate family support, professional mediation, and community awareness, while promoting the participation and involvement of people with YOD in the design of activities and support structures.

Finally, at a methodological level, the study confirms the value of spontaneous, participant-led conversations as a complementary source of knowledge to individual interviews, capable of capturing how experiences are expressed and negotiated in interaction.

Overall, the findings highlight the importance of recognizing people living with YOD as knowledgeable agents of their own experience, and of creating research conditions that allow their perspectives to emerge in their own terms, particularly through approaches that minimize researcher-imposed structures and enable participant-led expression.

## Data Availability

The original contributions presented in the study are included in the article/supplementary material, further inquiries can be directed to the corresponding author/s.
